# NFkB activation promotes immune activation in HTLV-I-associated myelopathy / tropical spastic paraparesis

**DOI:** 10.1186/1742-4690-8-S1-A117

**Published:** 2011-06-06

**Authors:** Matthew McCormick, Unsong Oh, Dibyadeep Datta, Richard Turner, Kathryn Bobb, Dileep Monie, Drago R Sliskovic, Jie Zhang, Jeffrey Meshulam, Steven Jacobson

**Affiliations:** 1 Neuroimmunology Branch, National Institute of Neurological Diseases and Stroke, National Institutes of Health, Bethesda, MD, USA; 2 Profectus Biosciences, Inc., Baltimore, MD, USA; 3 IDSC, Chelsea, MI, 48118, USA

## 

Evidence suggests that HTLV-I-induced immune activation plays a key role in the pathogenesis of HAM/TSP. The HTLV-I-encoded transactivating protein Tax is known to activate nuclear factor kappa B (NFkB), a key host signaling pathway regulating immune response, but the contribution of the NFkB pathway to the immune activation associated with HAM/TSP has yet to be fully defined. We examined NFkB activation in peripheral-blood mononuclear cells (PBMC) from subjects with HAM/TSP, and tested the effect of NFkB inhibition on key ex vivo correlates of immune activation in HAM/TSP.

We examined the role of NFkB activation during immune activation associated with HAM/TSP by using small molecule NFkB inhibitors, including a newly developed selective inhibitor of NFkB, PBS-1086.

NFkB activation was assessed in peripheral-blood mononuclear cells (PBMC) from subjects with HAM/TSP and in healthy donors (HD). Nuclear translocation of the NFkB RelA was significantly higher in PBMC from subjects with HAM/TSP compared to HD (p=0.032) following short-term (20 h) culture, indicating increased activation of the NFkB pathway in HAM/TSP. Treatment with the small molecule inhibitor PBS-1086 reduced NFkB activation (p<0.01). PBS-1086 reduced expression of CD25 and CD69 in HAM/TSP PBMC as well as phosphorylation of STAT5 in a dose-dependent manner (p<0.01 for all). PBS-1086 also inhibited spontaneous lymphoproliferation of HAM/TSP PBMC in a dose-dependent manner (p=0.0286). PBS-1086 treatment resulted in a mean proviral load reduction of 20% compared to untreated PBMC in a 72 h culture

These results indicate that NFkB activation plays a critical upstream role in the immune activation associated with HAM/TSP, and identify the NFkB pathway as a potential therapeutic target for immune modulation in HAM/TSP.

